# Determination of cell survival after irradiation via clonogenic assay versus multiple MTT Assay - A comparative study

**DOI:** 10.1186/1748-717X-7-1

**Published:** 2012-01-03

**Authors:** Karl Buch, Tanja Peters, Thomas Nawroth, Markus Sänger, Heinz Schmidberger, Peter Langguth

**Affiliations:** 1 Department of Pharmaceutical Technology and Biopharmaceutics, Johannes Gutenberg-University, Staudingerweg 5, 55099 Mainz, Germany; 2 Department of Radiooncology and Radiotherapy, University Medical Center, Johannes Gutenberg-University, Langenbeckstrasse 1, 55131 Mainz, Germany

## Abstract

For studying proliferation and determination of survival of cancer cells after irradiation, the multiple MTT assay, based on the reduction of a yellow water soluble tetrazolium salt to a purple water insoluble formazan dye by living cells was modified from a single-point towards a proliferation assay. This assay can be performed with a large number of samples in short time using multi-well-plates, assays can be performed semi-automatically with a microplate reader. Survival, the calculated parameter in this assay, is determined mathematically. Exponential growth in both control and irradiated groups was proven as the underlying basis of the applicability of the multiple MTT assay. The equivalence to a clonogenic survival assay with its disadvantages such as time consumption was proven in two setups including plating of cells before and after irradiation. Three cell lines (A 549, LN 229 and F 98) were included in the experiment to study its principal and general applicability.

## Background

Clonogenic and MTT assays are well-known tests for evaluation of chemoradiation studies and radiosensitivity [1-4]. Clonogenic assays are commonly used to investigate survival of irradiated cancer cells, whereas MTT assays are well known to study chemosensitivity [5] or toxicity [6] of drugs in human tumor cell lines. The assay is less common to study survival of cancer cells after irradiation, in particular when the MTT assay is performed for studying proliferation of treated cells.

The aim of this study is to compare the well-established clonogenic assay with an adapted version of the MTT proliferation assay to overcome limitations such as long duration of experiment, low sample throughput, limited error level and time-consuming counting of clones.

The MTT assay is based on the formation of dark-colored formazan dye by reduction of the tetrazolium salt MTT by metabolically active cells [7]. After some incubation, the water-insoluble formazan dye forms crystals, which can be dissolved in an organic solvent and the amount can be determined semi-automatically using a microplate reader. Absorbance readings are related to the number of cells [8] therefore providing the possibility to use the MTT assay as a proliferation assay to assess cell growth after irradiation. In the present study our adapted version of the MTT assay is compared to the clonogenic assay in order to open the possibility of replacing one by the other.

In the literature, several studies on comparability of MTT and clonogenic assay can be found. There, the MTT assay is done as a single-point assay after a defined time following treatment. In this case, much information about the growth behavior of the cells is lost (doubling time, lag phase, growth behavior etc.). In contrast, with our multiple MTT assay, we collect all those data and use them for more detailed interpretation.

## Materials and methods

### Cell line and Culture conditions

Experiments were carried out with A 549 cells (human NSCLC cell line), LN 229 cells (human glioblastoma cell line) and F 98 cells (glioblastoma cell line from rats). Cells were purchased from DSMZ (Braunschweig, Germany) and from ATCC (Manassas, USA). Cells were cultured in DMEM, supplemented with 10% fetal bovine serum (FBS), 10 mg/ml antibiotics (penicillin and streptomycin) at 37°C with 5% CO_2 _in humidified air. Cell media were obtained from Invitrogen (Darmstadt, Germany). All other chemicals were purchased from Roth (Karlsruhe, Germany). Cell concentrations in the culture were adjusted to allow for exponential growth.

### Experiment setup

The irradiation cell culture experiment was based on literature methods [9]. Two essentially different ways of performing this study can be carried out: Plating of cells before or after irradiation. Plating before irradiation is often used as a screening method of sensitivity and efficiency of different treatments. Plating after treatment is used especially to investigate cell damage repair [9]. Both methods were integrated in this study.

#### Plating after irradiation

For irradiation experiments, cells were grown in flasks (25 cm^2^) to reach 90% confluence and they were completely filled with DMEM to avoid artifacts by irradiation through air layers. Treated cells were harvested the next day using trypsinization, counted and a specific number of cells (500 and 250 cells) was plated in petri dishes in triplicate for clonogenic assay. The multiple MTT assay was performed using 96-well-plates with 4,000 and 2,000 cells (for A 549) per well, respectively. Plating density for LN 229 was adapted to the size of the cells.

#### Plating before irradiation

Cells from a stock culture were plated following appropriate dilutions (100 and 50 cells/well for A 549, adapted to the smaller size of a well compared to a petri dish) into 6-well-plates as well as into 96-well-plates (4,000 and 2,000 cells/well for A 549) for multiple MTT assay and allowed to attach overnight. Irradiation treatment was performed on the next day. Plating density for LN 229 and F 98 was adapted to the size of the cells.

#### Proliferation development

Dishes and multi-well-plates were placed in an incubator. Dishes and 6-well-plates were left there for 9 days until large clones (> 1 mm) were formed (50 cells or more). Staining of colonies was done as described below.

#### Plating density

Commonly, plating densities for clonogenic assays were adapted paying attention to the situation that the endpoint analysis of this assay is determined optically by counting clones. Therefore, plating density must not be too high or clones will coalesce and counting of single colonies will become impossible. This fact does not play a role in our multiple MTT assay where analysis is done photometrically. Here, the only limitation to the validity of the results of the MTT assay is, when confluence has been reached or in cases where cell growth has stopped due to depletion of medium. Previous experiments showed that plating densities can vary from 500 cells/well up to 20,000 cells/well (data not shown here).

### Irradiation procedure

Irradiation was done using a 6 MV linear accelerator Siemens MD-2 using a clinically calibrated irradiation field of 20 × 20 cm. The irradiation dose varied between 0 and 15 Gy. For plating after irradiation, cell culture flasks were completely filled with medium and irradiated horizontally in upright position with RW3-plastic material in front of the flasks and behind as phantom simulating tissue. For plating before irradiation, the plates were placed horizontally and covered with RW3-plates as well.

### Fixation and staining of colonies

Medium was removed in both dishes and plates, cells were rinsed with PBS. Fixation and staining of clones was done with a mixture of 0.5% crystal violet in 50/50 methanol/water for 30 min. Dishes were rinsed with water and left for drying at room temperature. Counting of clones was done on the following day.

### Multiple MTT assay

The protocol is adapted from literature methods [8]. Briefly, DMEM was supplemented with 100 μl of MTT (3-(4,5-Dimethylthiazol-2-yl)-2,5-diphenyltetrazolium bromide) reagent (c = 0.5 g/l) to each well and incubated for 30 min at 37°C. Thereafter MTT solution was removed. After addition of 180 μl of DMSO the plates were incubated for 15 min at 37°C to dissolve the formazan crystals. Absorbance readings of DMSO extracts were performed at 560 nm with reference of 690 nm using a Tecan Infinite F 200 microplate reader (Crailsheim, Germany).

## Results and discussion

### Clonogenic assay

After counting clones, plating efficiency (PE) and survival fraction (SF) can be calculated using the following equations [9]:

(1) $$PE=\frac{\#\;of\;colonies\;formed}{\#\;of\;cells\;seeded}\times 100\%$$

(2) $$SF=\frac{\#\;of\;colonies\;formed\;after\;irradiation}{\#\;of\;cells\;seeded\times PE}$$

PE was about 64% for both methods (plating before and after irradiation) of this study. SF data of all investigated cell lines are shown in Table 1. The survival curves which have been obtained using different plating densities were virtually identical and therefore were averaged.

**Table 1:** Clonogenic Assay: Surviving Fraction (SF) of irradiated cells (n = 3 for each experiment); n.d. = not determined

	plating after irradiation				plating before irradiation			
**A 549**	**250 cells**	**500 cells**	**mean value**	**S.D**.	**50 cells**	**100 cells**	**mean value**	**S.D**.

0 Gy	100.0%	100.0%	100.0%		100.0%	100.0%	100.0%	
1 Gy	67.9%	66.3%	67.1%	1.1%	97.7%	87.2%	92.5%	7.4%
2 Gy	62.3%	60.8%	61.6%	1.0%	90.7%	75.8%	83.3%	10.5%
4 Gy	22.6%	24.7%	23.7%	1.5%	55.8%	34.6%	45.2%	15.0%
6 Gy	n.d.				25.6%	19.0%	22.3%	4.7%
8 Gy	5.0%	4.1%	4.6%	0.6%	16.3%	10.4%	13.4%	4.1%
15 Gy	3.4%	0.0%	1.7%	2.4%	2.3%	1.9%	2.1%	0.3%

**LN 229**	**250 cells**	**500 cells**	**Mean value**	**S.D**.	**100 cells**	**200 cells**	**Mean value**	**S.D**.

0 Gy	100.0%	100.0%	100.0%		100.0%	100.0%	100.0%	
1 Gy	90.5%	90.2%	88.9%	3.1%	109.0%	89.5%	99.5%	16.7%
2 Gy	77.0%	60.3%	67.7%	2.2%	38.8%	45.0%	41.9%	12.7%
4 Gy	20.3%	21.1%	20.6%	0.7%	12.9%	14.1%	13.5%	2.4%
6 Gy	n.d.				1.6%	3.7%	2.6%	1.7%
8 Gy	1.7%	1.7%	1.7%	0.1%	0.0%	0.0%	0.0%	0.0%
15 Gy	0.0%	0.0%	0.0%	0.0%	0.0%	0.0%	0.0%	0.0%

**F 98**					**75 cells**	**100 cells**	**Mean value**	**S.D**.

0 Gy	n.d.				100.0%	100.0%	100.0%	
1 Gy					74.1%	57.4%	65.8%	15.4%
4 Gy					33.3%	41.0%	37.2%	7.7%
6 Gy					33.3%	28.7%	31.0%	8.8%

### Multiple MTT assay

**Figure 1:**
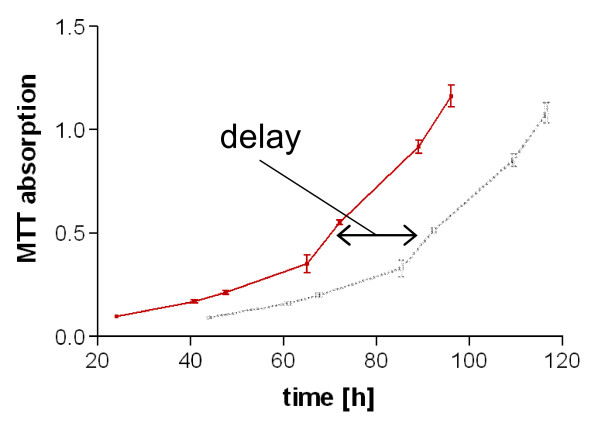
**Irradiation induced delay of a cell growth curve: cells were irradiated at t = 20 h after seeding, red curve: control at 0 Gy, grey curve: irradiated cells with a delay of about 20 h in growth, used as basis for calculation of multiple MTT survival**.

MTT assay was done once every day with one column of each multi-well-plate to follow up exponential growth of cells. MTT readings are proportional to the number of cells in-vitro, at least in the phase of exponential growth. This fact is described in the literature [8] and was verified in our lab (data not shown). For calculation of the proliferation survival, only the early exponential phase of cell growth is used in the following equation (a derived equation, developed by ourselves):

(3) $$Survival\;=\;2^{-\;\frac{t_{delay}}{t_{doubling\;time}}}$$

t_doubling time _= the time period required for a quantity of cells to double

t_delay _= the time period to reach specific absorption value of control vs. irradiated cells (graphically shown in Figure 1)

Survival results are shown in Table 2.

**Table 2:** Multiple MTT assay: Survival of irradiated cells (n = 8 for each experiment); n.d. = not determined

	plating after irradiation				plating before irradiation			
**A 549**	**2000 cells**	**4000 cells**	**mean value**	**S.D**.	**2000 cells**	**4000 cells**	**mean value**	**S.D**.

0 Gy	100.0%	100.0%	100.0%		100.0%	100.0%	100.0%	
1 Gy	64.3%	63.0%	63.7%	0.9%	102.6 &	87.5%	95.0%	10.7%
2 Gy	73.4%	56.5%	65.0%	11.9%	102.5%	88.0%	95.3%	10.2%
4 Gy	28.6%	34.5%	31.6%	4.2%	65.6%	67.6%	66.7%	1.4%
6 Gy	n.d.				45.9%	76.8%	61.4%	21.8%
8 Gy	3.2%	13.4%	8.3%	7.3%	27.6%	43.1%	35.3%	11.0%
15 Gy	0.0%	0.1%	0.1%	0.1%	26.3%	31.3%	28.8%	3.6%

**LN 229**	**2500 cells**	**4000 cells**	**Mean value**	**S.D**.	**2500 cells**	**5000 cells**	**Mean value**	**S.D**.

0 Gy	100.0%	100.0%	100.0%		100.0%	100.0%	100.0%	
1 Gy	83.2%	92.3%	87.7%	6.6%	81.9%	87.6%	84.8%	3.9%
2 Gy	62.4%	70.9%	66.7%	7.8%	87.7%	79.9%	83.8%	5.8%
4 Gy	43.0%	50.9%	47.0%	8.5%	63.6%	80.7%	72.2%	3.7%
6 Gy	n.d.				30.4%	57.3%	43.9%	5.1%
8 Gy	27.6%	39.8%	33.7%	7.2%	27.1%	48.7%	37.9%	2.6%
15 Gy	15.0%	19.6%	17.3%	5.2%	12.4%	30.7%	21.6%	1.9%

**F 98**					**2500 cells**	**4000 cells**	**Mean value**	**S.D**.

0 Gy	n.d.				100.0%	100.0%	100.0%	
1 Gy					93.7%	97.1%	95.4%	7.5%
4 Gy					69,7%	65.6%	67.6%	5.0%
6 Gy					46.2%	54.9%	50.6%	4.4%
8 Gy					28.7%	38.7%	33.7%	3.8%
15 Gy					6.9%	9.7%	8.4%	1.2%

**Figure 2:**
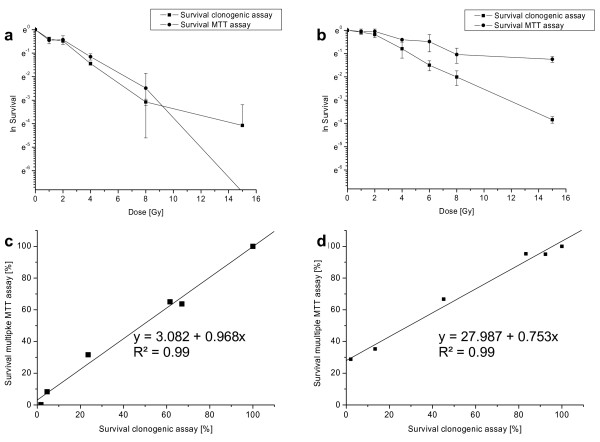
**Survival data (natural logarithm) and correlation of clonogenic and multiple MTT assay for A 549 cells: a & c: plating after irradiation; b & d: plating before irradiation (cells were irradiated 20 h after seeding)**.

In Figure 2a and 2b the survival data for A 549 cells of clonogenic and multiple MTT assay are plotted against irradiation dose. The correlation of both assays is presented in Figure 2c and 2d (R^2 ^= 0.99 for both). As expected, the survival curves of both assays show the same characteristics: the survival depends on the dose of irradiation and an increase of dose leads to a decrease in survival, approaching zero in case of plating the cells after irradiation. Clonogenic and multiple MTT assay show very similar curve progression, while the multiple MTT assay gives slightly higher activity results. This fact may be explained by the presence of cells which are alive and show metabolic activity, but poor or no cell proliferation. Those cells gain more influence in the outcome of survival in case of the multiple MTT assay. If the plating of cells is done before irradiation, metabolic cell activity during the following assay may vary significantly. Cells with low metabolic activity and slow proliferation or cells which ceased to proliferate, are excluded from the assay by washing and trypsinization, when the plating is done after irradiation. However, as shown in Figure 2d, the correlation between clonogenic and multiple MTT assay is still very good and linear.

**Figure 3:**
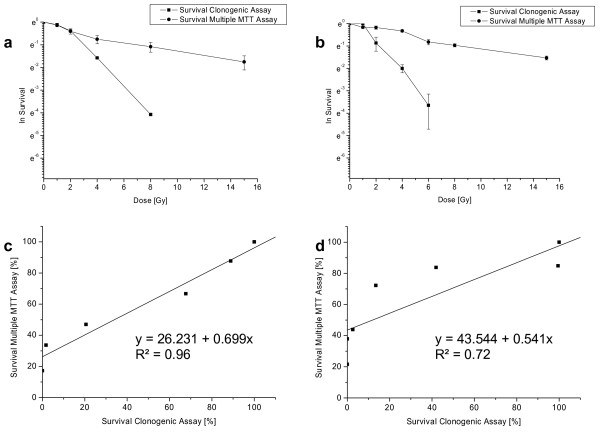
**Survival data (natural logarithm) and correlation of clonogenic and multiple MTT assay for LN 229 cells: a & c: plating after irradiation; b & d: plating before irradiation (cells were irradiated 20 h after seeding)**.

To prove general applicability of our method, further cell lines were included in this study. Survival data for LN 229 cells of clonogenic and multiple MTT assay are shown in Figure 3a and 3b as a plot of survival against irradiation dose. The correlation of the two assays is shown in Figure 3c and 3d (R^2 ^= 0.96 and R^2 ^= 0.72, respectively). Curve progression of clonogenic survival is similar to MTT survival, while clonogenic survival decreased even more than MTT survival. Correlation of the two assays is still acceptable for plating after irradiation (R^2 ^= 0.96), for plating before irradiation the correlation quality decreased (R^2 ^= 0.72).

**Figure 4:**
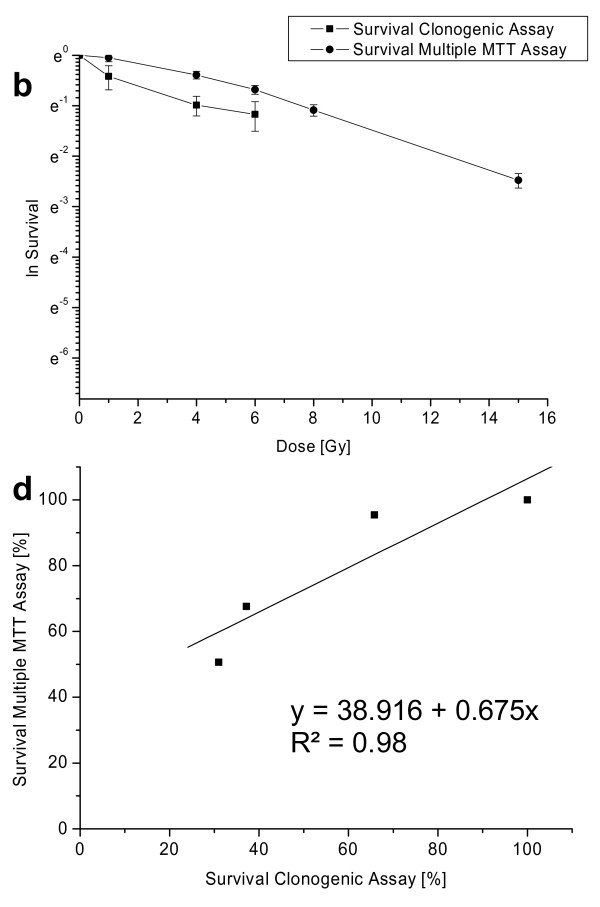
**Survival data (natural logarithm) and correlation of clonogenic and multiple MTT assay for F 98 cells: b & d: plating before irradiation (cells were irradiated 20 h after seeding)**.

For the setup 'plating before irradiation' A 549 cells showed a very good correlation, LN 229 reduced correlation quality. Therefore, we included a third cell line (F 98) in this study to prove the general applicability. Survival data are shown in Figure 4b, correlation data are shown in Figure 4d (R^2 ^= 0.98) as a good linearity between the two assays.

The biggest advantage of performing the multiple MTT assay is that, due to the fact that our assay is not only a single-point determination of survival, information about growth performance can be acquired easily. Degrees of direct cell death, lag phase after irradiation and changes in doubling-time can be measured by assay-time-points before and after irradiation. Even if growth becomes exponential again after the lag phase due to irradiation treatment, doubling-time maybe not the same as in control [10] in our study but increases following irradiation with high doses.

## Conclusion

Our results suggest that the multiple MTT assay can be a surrogate of the clonogenic assay in order to determine survival of irradiated tumor cells. Requirements for correct interpretation of results are on the one hand linearity of cell number and MTT reading and on the other hand, a sufficient number of time points over several cell generations in order to predict growth behavior accurately. The main and only disadvantage of this assay is that the survival may be overestimated at high radiation doses in the case of plating before irradiation. However, correlation of multiple MTT and clonogenic assay is good and trends can be determined anyway; an important fact when different treatments under equal irradiation doses are performed. The main advantage of the multiple MTT assay presented is the opportunity to obtain precise survival data for high sample throughput in less time and with less effort than with the conventional colony assay.

In order to establish the multiple MTT assay instead of a clonogenic assay it is expedient to examine the transferability of the method first. An experimental setup reduced to two or three radiation doses should be sufficient to verify the comparability.

## Competing interests

The authors declare that they have no competing interests.

## Authors' contributions

KB and TP designed the experimental setup and carried out the cell culture experiments. MS calculated the irradiation parameters and operated the linear accelerator, TN developed the multiple MTT assay model, HS allocated the irradiation facility, HS and PL conceived of the study, KB drafted the manuscript. All authors read and approved the final manuscript.
